# Stated job preferences of three health worker cadres in Ethiopia: a discrete choice experiment

**DOI:** 10.1093/heapol/czab081

**Published:** 2021-07-27

**Authors:** Shyam Lamba, Nikita Arora, Dorka Woldesenbet Keraga, Abiyou Kiflie, Birkety Mengistu Jembere, Della Berhanu, Mehret Dubale, Tanya Marchant, Joanna Schellenberg, Nasir Umar, Abiy Seifu Estafinos, Matthew Quaife

**Affiliations:** Faculty of Public Health and Policy, London School of Hygiene and Tropical Medicine, Keppel Street, London WC1E 7HT, UK; Faculty of Public Health and Policy, London School of Hygiene and Tropical Medicine, Keppel Street, London WC1E 7HT, UK; School of Public Health, Addis Ababa University, P.O. Box: 9086, Addis Ababa, Ethiopia; Institute for Healthcare Improvement, Addis Ababa, Ethiopia; Institute for Healthcare Improvement, Addis Ababa, Ethiopia; Faculty of Infectious and Tropical Diseases, London School of Hygiene and Tropical Medicine, Keppel Street, London WC1E 7HT, UK; Faculty of Infectious and Tropical Diseases, London School of Hygiene and Tropical Medicine, Keppel Street, London WC1E 7HT, UK; Faculty of Infectious and Tropical Diseases, London School of Hygiene and Tropical Medicine, Keppel Street, London WC1E 7HT, UK; Faculty of Infectious and Tropical Diseases, London School of Hygiene and Tropical Medicine, Keppel Street, London WC1E 7HT, UK; Faculty of Infectious and Tropical Diseases, London School of Hygiene and Tropical Medicine, Keppel Street, London WC1E 7HT, UK; School of Public Health, Addis Ababa University, P.O. Box: 9086, Addis Ababa, Ethiopia; Faculty of Public Health and Policy, London School of Hygiene and Tropical Medicine, Keppel Street, London WC1E 7HT, UK

**Keywords:** Discrete choice experiment, job preferences, stated preference, human resources for health, Ethiopia, health extension workers, care providers, non-patient-facing staff

## Abstract

Attracting, training and retaining high-quality health workers are critical for a health system to function well, and it is important to know what health workers value in their roles. Many studies eliciting the labour market preferences of health workers have interviewed doctors or medical students, and there has been little research on the job preferences of lower-skilled cadres such as community health workers, mid-skilled clinical care staff such as nurses and midwives, or non-patient facing staff who manage health facilities. This study estimated the job preferences of public health sector community health extension workers (HEWs), care providers including nurses and midwives, and non-patient-facing administrative and managerial staff in Ethiopia. We used a discrete choice experiment to estimate which aspects of a job are most influential to health worker choices. A multinomial logistic regression model estimated the importance of six attributes to respondents: salary, training, workload, facility quality, management and opportunities to improve patient outcomes. We found that non-financial factors were important to respondents from all three cadres: e.g., supportive management [odds ratio (OR) = 2.96, *P*-value = 0.001] was the only attribute that influenced the job choices of non-patient-facing administrative and managerial staff. Training opportunities (OR = 3.45, *P*-value < 0.001), supportive management (OR = 3.26, *P*-value < 0.001) and good facility quality (OR = 2.42, *P*-value < 0.001) were valued the most amongst HEWs. Similarly, supportive management (OR = 3.22, *P*-value < 0.001), good facility quality (OR = 2.69, *P*-value < 0.001) and training opportunities (OR = 2.67, *P*-value < 0.001) influenced the job choices of care providers the most. Earning an average salary also influenced the jobs choices of HEWs (OR = 1.43, *P*-value = 0.02) and care providers (OR = 2.00, *P*-value < 0.001), which shows that a combination of financial and non-financial incentives should be considered to motivate health workers in Ethiopia.

**Key messages**

- This study used a discrete choice experiment to estimate which aspects of a job are most influential to health worker choices in Ethiopia, including health extension workers (HEWs), care providers such as nurses and midwives, and non-patient-facing administrative and managerial staff.
- We found that non-financial factors were important to respondents from all three cadres: e.g., a supportive management style was found to be the most important attribute across all cadres in Ethiopia including HEWs, care providers and non-patient-facing administrative and managerial staff.
- Earning an average salary also influenced the job choices of HEWs and care providers; however, other attributes were more important including good facility quality and 5 days of training per year.

## Introduction

Motivation and retention of health workers are a key challenge in low- and middle-income countries (LMICs)—some of which face severe human resource shortages (Chen *et al.*, 2004; World Health Organization, 2006; 2016). In many countries, it is presumed that health workers are motivated by an overall desire to improve patient outcomes (Lindelow and Serneels, 2006). However, in many LMIC settings, including Ethiopia, the health labour market is characterized by high attrition, geographical inequity and low morale (Alebachew and Waddington, 2015; Federal Democratic Republic of Ethiopia Ministry of Health, 2015). An understanding of job preferences can help policymakers better align incentives and retain a motivated workforce (Lindelow and Serneels, 2006; Lagarde and Blaauw, 2009; Blaauw *et al.*, 2013). While few studies have quantitatively explored the trade-offs health workers make when choosing between job attributes, discrete choice experiments (DCEs) have become a popular method in recent years to estimate how health workers make decisions in the labour market (Mandeville *et al.*, 2014). The majority of DCE studies for health workforce policy have focused on the labour preferences of qualified doctors and medical students (Mandeville *et al.*, 2014). A few studies have analysed the preferences of mid-level health workers, such as clinical officers in Tanzania and medical assistants in Lao (Kolstad, 2011; Jaskiewicz *et al.*, 2012). However, there has been little research conducted on the job preferences of lower-skilled cadres such as community health workers, mid-skilled clinical care staff such as nurses and midwives, or non-patient-facing administrative and managerial staff that largely manage health facilities (Mandeville *et al.*, 2014).

This study aims to estimate and compare the job preferences of three cadres working in the public health sector in Ethiopia, including community health workers called health extension workers (HEWs), care providers (i.e. clinicians) such as nurses and midwives, and non-patient-facing administrative and managerial staff. We used a DCE to estimate which aspects of a job are most influential to health worker choices.

## Methods

### Study context

**Figure 1.**
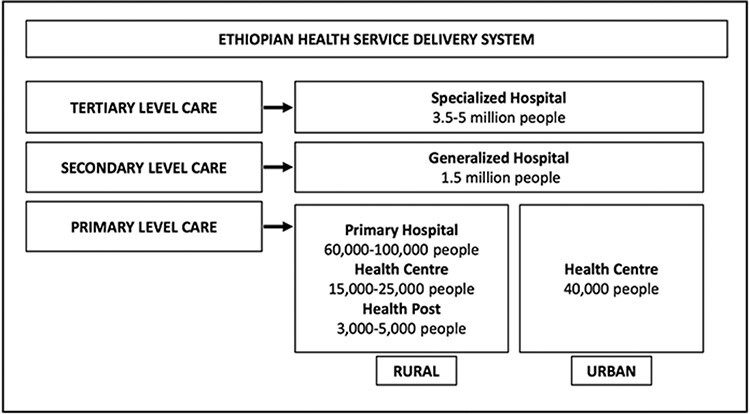
Ethiopia health system (reproduced with permission from Alebachew and Waddington, 2015)

The study was conducted in Ethiopia, which is divided into nine regions and two city administrations. In each region, woredas (districts) are administrative units, managed by decentralized councils of elected members (Workie and Ramana, 2013). The Ethiopian healthcare delivery system, referred to as the three-tiered system, provides healthcare services to people at primary, secondary and tertiary levels (Alebachew and Waddington, 2015). The primary level, where this study operated, consists of three service delivery points: health post, health centre and primary hospital (Figure 1).

The primary healthcare workforce includes facility- and community-based health workers supported by non-patient-facing management and administrative staff (World Health Organization, 2020). HEWs are assigned to health posts as salaried government employees following a 12-month training programme (Assefa *et al.*, 2019). They are usually hired as Level 3 health workers and have the opportunity to upskill and be redeployed to higher positions in the health system after taking a competitive exam. The average attrition rate of HEWs is around 3% per year with some evidence suggesting a continuing rise since the start of the Health Extension Programme (HEP) (Arora *et al.*, 2020). Evidence suggests that around 70% of HEWs have a desire to upgrade as a nurse, although to what extent that is possible is not clear (Teklehaimanot *et al.*, 2007). Yet, factors affecting the retention of HEWs are largely due to non-material factors, such as community acceptance or acknowledgement from supervisors and senior managers (Arora *et al.*, 2020). In contrast, factors affecting the retention of public sector nurses and midwives are a mix of financial and material incentives (e.g. better remuneration and improved infrastructure), whereas one recent study in Ethiopia found that around 50% of nurses and midwives intended to leave their current job in the following year (Ayalew *et al.*, 2015; Muluneh *et al.*, 2021). Some evidence also suggests that access to a large labour market with competing salaries and infrastructure quality (e.g. in non-governmental organizations, the private sector and international labour market) was also another reason for the high turnover of government-employed nurses and midwives in Ethiopia (Mariam, 2013; Ayalew *et al.*, 2015; Muluneh *et al.*, 2021).

To our knowledge, there is no published evidence of retention among non-patient-facing staff or the factors influencing retention of non-patient-facing staff in public sector health facilities, despite their essential role in overseeing the functioning of the healthcare delivery system.

### Sample and data collection

The DCE was embedded within a baseline data collection of a survey conducted as part of the process evaluation of a quality improvement (QI) programme implemented by the Institute of Healthcare Improvement and the Ethiopian Federal Ministry of Health. At the time of data collection, no participants had been exposed to the QI programme.

Data were sampled from four out of the nine Ethiopian regions for this study. Using a random number generator, we randomly selected one QI programme woreda per region from Oromia, Amhara, Southern Nations, Nationalities, and Peoples’ Region (SNNPR) and Tigray. We added one additional randomly selected woreda in Amhara since the first randomly selected woreda in Amhara had too few health facilities to reach the sample size. We further purposively sampled two additional woredas from Oromia and SNNPR (Bunno Bedelle and Chencha, respectively) where other evaluative work was also taking place. For each of the seven QI programme woreda chosen for data collection, we chose one matched woreda from the same region which was not subject to QI activities, resulting in 14 woredas in total. The woredas were matched using service utilization data from the last three Ethiopia Demographic and Health Surveys (2005; 2011; 2016).

In each woreda, we sought to interview 30 participants across a range of health worker and management cadres, where the latter included facility heads alongside woreda and regional health office managers. Senior non-patient-facing staffs in each woreda were not randomly sampled due to their small number, but staff at primary hospitals, health centres and health posts were randomly sampled. The heads or clinical directors of each woreda (one), primary hospital (one) and health centre (three) were interviewed. Four maternal and child health clinical care providers and two from each health centre were interviewed in the hospital. One HEW was interviewed from each health post under each health centre.

The baseline survey was conducted from April 15 to May 10, 2018. We obtained a stratified random sample of 401 workers in the Ethiopian health system across three cadres: 202 (50.4%) HEWs, 155 (38.7%) care providers (including 100 midwives) and 43 (10.7%) non-patient-facing administrative and managerial staff. A team of seven trained research assistants from the authors’ institute implemented a face-to-face survey administered in English, Amharic and Oromifa languages using Open Data Kit (https://opendatakit.org) software on tablet computers. Informed consent was obtained from all participants before data were collected.

### DCE development and design

The attributes and findings of published DCEs conducted in east Africa were analysed to inform the development of our DCE (Mangham and Hanson, 2008; Blaauw *et al.*, 2010; Kolstad, 2011; Rockers *et al.*, 2012; Mandeville *et al.*, 2014; 2016). A shortlist of potential attributes was generated and reduced to six using the findings of a qualitative study conducted 1 year previously, assessing the motivation of HEWs in Ethiopia (Tesfaye, 2017). As there is some debate on the use of text or images to represent attributes and levels in DCEs, we opted to display choice tasks as text since pictures can convey their own meanings, sometimes different from the text, which can cause confusion (Veldwijk *et al.*, 2015).

**Figure 2.**
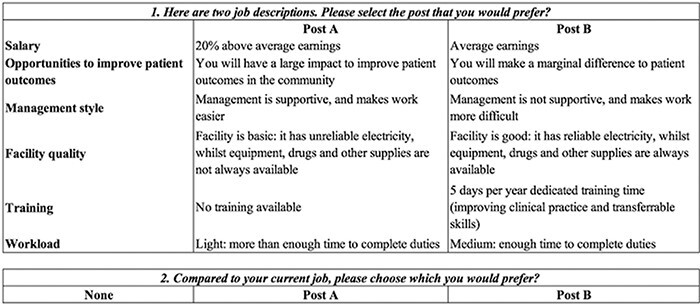
Example of choice task presented to study participants

We displayed two job profiles in each choice task using an unlabelled design where each alternative represents a generic health worker’s job, within which all selected characteristics change as prescribed by the statistical design. Participants were asked the following question: ‘Here are two jobs described by some of their characteristics. Compared to your current job, please choose which you would prefer’. To increase realism and allow for the estimation of unconditional demand, a generic opt-out alternative was included to represent their current job. The final six attributes of the DCE and their levels are shown in Table 1, and Figure 2 shows an example of how choice tasks are presented to respondents. The final design incorporated seven choice tasks.

**Table 1.** Choice experiment attributes and levels

Attribute	Levels
Salary	20% below average; Average earnings; 20% above average
Opportunities to improve health	Your work will have a ‘large’ impact on improving health in the local community; Your work will have a ‘small’ impact on improving health in the local community
Management style	Management ‘is supportive’ and makes work ‘easier’; Management is ‘not supportive’ and makes work ‘more difficult’
Office quality	Your workplace is ‘good’: it has ‘reliable’ electricity and other services, and supplies are ‘always available’; Your office is ‘basic’: it has ‘unreliable’ electricity, whilst supplies you need are ‘not always available’
Training	No training available; 5 days per year dedicated training time (improving work-related and transferable skills); 10 days per year dedicated training time (improving work-related and transferable skills)
Workload	‘Light’: more than enough time to complete duties; ‘Medium’: enough time to complete duties; ‘Heavy’: barely enough time to complete duties

The DCE was piloted among 19 woreda health office staff in December 2017. No changes were made to the DCE between piloting and the final survey as it was understood well by participants. Priors from analysis of pilot data (*n* = 19) were used in NGENE software (http://www.choice-metrics.com/) to generate a single D-error minimizing design with 10 tasks, which avoided dominant or duplicated alternatives with the aim of improving precision in the final model estimates.

### Main effect models

Choices were modelled based on McFadden’s random utility theory (McFadden, 1973). This assumes that respondent $n$ will choose alternative $j$ in choice set $c$ if that alternative provides the most satisfaction out of all other alternatives. This is shown in the following Equation (1):

(1) \begin{equation*}{U_{{njc}}} = {V_{{njc}}} + {\varepsilon _{{njc}}}\end{equation*}

where ${U_{njc}}$ is the utility function of individual $n$ from choosing alternative $j$ in choice set $c$; ${V_{njc}}$ signifies the observable element for choosing alternative $j$ and ${\varepsilon _{njc}}$ represents the random, unobservable element for choosing alternative $j$. Equation (2) represents the ‘indirect utility function’ of Equation (1).

(2) \begin{equation*}{V_{\textit{njc}}} = {X_{{njc}}}\beta + {\varepsilon _{{njc}}}\end{equation*}

where ${X_{{njc}}}{\beta }$ represents a linear specification of the DCE attributes, as shown in Equation (3). The probability of choosing alternative $j$ is captured by a set of observable attributes, ${X_{njc}}$, which takes the following form:

(3) \begin{align*}{X_{{njc}}}{B_j} =& \,{\beta _0} + {\beta _1}{Salar}{{y}_j} + {\beta _2}{Impac}{{t}_j} + {\beta _3}{Managemen}{{t}_j} \nonumber\\&+ {\beta _4}{Facilit}{{y}_j} + {\beta _5}{Trainin}{{g}_j} + {\beta _6}{Workloa}{{d}_j}\end{align*}

where ${\beta _0}$ represents the constant, and salary, impact, management, facility, training and workload were the attributes used in the DCE. This is underpinned by Lancaster’s consumer behaviour theory, which assumes that utility is derived from the characteristics of a certain good (Cascetta, 2009; Lagarde and Blaauw, 2009; Mandeville *et al.*, 2014; Lancsar *et al.*, 2017).

Using specifications from Equation (3), Equation (1) was estimated using a multinomial logit (MNL) model, which generally assumes that the stochastic term, ${\varepsilon _{njc}}$, is independently and identically distributed (IID). The IID assumption assumes that unobserved effects are not related in any systematic way with the observed effects and in practice assumes preference homogeneity across individuals (Hensher *et al.*, 2005). Standard errors were clustered at the facility level, relaxing the IID assumption by allowing for intra-group correlation. This meant that observations from the same facility were not independent, but observations remained independent across groups (Lancsar *et al.*, 2017; StataCorp, 2019).

### Preference heterogeneity

Preference heterogeneity was explored by cadre. We conducted a subgroup analysis of the main effects by running separate regressions on three sub-groups of health workers to reveal any variation in preferences. This included HEWs, care providers such as nurses and midwives, and non-patient-facing administrative and managerial staff. The same attributes were used across the three cadres to allow us to make comparisons between each cadre’s trade-offs. Individual characteristics were not adjusted for in the model due to the small sample size of some of the sub-groups and the presence of multicollinearity.

### Estimating the MNL model

Stata 15 was used to estimate the MNL models, and odds ratios (ORs) used to estimate the relative importance of each attribute; the attributes were dummy coded and standard errors were clustered at the facility level. Utility was estimated as a measure of choice. ORs that are larger than one indicate positive impact on utility whereas those below one indicate disutility attached to the attribute level. Face validity was assessed by checking if ORs were of the expected sign (de Bekker‐Grob *et al.*, 2012). Additional robustness checks were carried out to check if the results changed by adjusting the standard errors at the individual level or removing the cluster adjustment altogether. A goodness-of-fit model was estimated using log pseudolikelihood and pseudo *R*-squared (Hauber *et al.*, 2016). Only ORs that are statistically significant at either the 5% or 1% level are reported.

## Results

### Descriptive statistics

Table 2 presents the characteristics of the DCE respondents. In total, 401 respondents were interviewed but 11 had missing data so were dropped from the study. The final dataset comprised 390 respondents in total, including 198 HEWs, 149 care providers consisting largely of nurses and midwives, and 43 non-patient-facing administrative and managerial staff. Around 73% (283/390) of respondents were female, 89% (347/390) were patient facing and the mean age was 28 years. Over 51% (198/390) of those surveyed were HEWs who were comparatively less qualified with an average gross salary below the whole sample average [Ethiopian Birr (ETB) 3291 per month which was equivalent to 119 US dollar (USD)]. Care providers were the next largest group surveyed, comprising around 38% (149/390) of the whole sample. Around 67% (100/149) of care providers were trained as midwifery professionals and around 15% (22/149) were trained as nursing professionals. Non-patient-facing administrative and managerial staff had the highest qualifications amongst the three sub-groups, of whom 50% (22/43) had a bachelors’ degree and earned a gross salary above the whole sample average (ETB 5669 per month which was equivalent to 206 USD). Overall, 35% (135/390) of respondents were from SNNPR, 26% (102/390) from Amhara, 27% (104/390) from Oromia and 13% (49/390) from Tigray.

**Table 2.** Characteristics of DCE respondents

Characteristics	HEWs (*N* = 198)	Care providers^a^ (*N* = 149)	Non-patient facing^b^ (*N* = 43)
Age:			
Mean age in years (SD, *N*)	27 (4, 198)	28 (5, 149)	30 (5, 43)
Gender:			
% Female (*N*)	99 (196)	52 (78)	21 (9)
Region:			
% Amhara (*N*)	26 (52)	30 (45)	12 (5)
% Oromia (*N*)	25 (49)	30 (45)	23 (10)
% SNNPR (*N*)	41 (82)	26 (38)	35 (15)
% Tigray (*N*)	8 (15)	14 (21)	30 (13)
Facility type:			
% Woreda health office (*N*)	0 (0)	0 (0)	16 (7)
% Hospital (*N*)	11 (22)	7 (10)	2 (1)
% Health centre (*N*)	34 (68)	55 (82)	37 (16)
% Health post (*N*)	55 (108)	38 (57)	44 (19)
No. of years working in health system:			
% Less than 1 year (*N*)	12 (24)	13 (20)	7 (3)
% 1–2 years (*N*)	5 (10)	13 (19)	5 (2)
% 2–4 years (*N*)	7 (14)	23 (34)	9 (4)
% More than 4 years (*N*)	76 (150)	51 (76)	79 (34)
Highest qualification:			
% School certificate (*N*)	20 (39)	1 (2)	5 (2)
% Diploma (*N*)	48 (95)	62 (92)	40 (17)
% Bachelors’ degree (*N*)	3 (6)	36 (53)	51 (22)
% Masters and above (*N*)	0 (1)	1 (2)	5 (2)
% Other qualifications (*N*)	29 (57)	0 (0)	0 (0)
Training background:			
% Generalist (non-specialist) medical doctor (*N*)	0 (0)	4 (6)	5 (2)
% Health officer (*N*)	0 (0)	10 (15)	28 (12)
% Nursing training (*N*)	10 (19)	15 (22)	16 (7)
% Midwifery training (*N*)	2 (3)	67 (100)	7 (3)
% HEW training (*N*)	87 (173)	0 (0)	14 (6)
% Other (*N*)	2 (3)	4 (6)	30 (13)
Gross salary:			
Average in ETB (SD, *N*)	3,291 (882, 198)	4185 (1711, 149)	5669 (2152, 43)
Average in USD^c^	119	152	206
Median in ETB [interquartile range (IQR)]	3137 (2654–4,062)	3,579 (3137–4,446)	5294 (4085–6580)
Median in USD (IQR)^c^	114 (96–148)	130 (114–161)	192 (148–239)
% Salary not increased in last 12 months (*N*)	75 (149)	66 (99)	58 (25)
% Received salary on time (*N*)	87 (172)	85 (126)	95 (41)

a Care providers includes trained clinicians such as midwives, nurses, health officers and medical doctors.

b Non-patient-facing staff includes administrative and managerial (non-clinical) staff, such as chief medical directors, Woreda health office head, CEO, public health staff and managers.

c Exchange rate as at April 30, 2018 (1 ETB = 0.0363 USD) via https://www.exchangerates.org.uk/historical/ETB/30_04_2018.

Table 3 presents the characteristics of the respondents’ current role. For example, around 50% (195/390) of respondents thought that they had an average salary relative to their colleagues. Over 92% (360/390) considered their work to have a large impact on improving health in the community, and over 87% (338/390) agreed that the facility quality was basic with unreliable electricity and supplies. Furthermore, 63% (245/390) reported that they had a heavy workload with barely enough time to complete duties and 42% (62/149) of care providers reported that their management was not supportive. Amongst the six attributes, 69% (125/180) of the sample reported that they are most motivated by opportunities to improve health outcomes. However, workload (29%), management style (22%) and salary (22%) were reported as the factors that demotivate health workers the most.

**Table 3.** Attribute characteristics based on view of DCE respondents

Characteristics	HEWs (*N* = 198)	Care providers (*N* = 149)	Non-patient facing (*N* = 43)
Salary:			
% Paid less than average relative to colleagues (*N*)	47 (93)	44 (66)	47 (20)
% Paid about average relative to colleagues (*N*)	50 (98)	52 (78)	44 (19)
% Paid more than average relative to colleagues (*N*)	3 (7)	4 (5)	9 (4)
Impact of work:			
% Agree that their work has a large impact (*N*)	94 (187)	88 (131)	98 (42)
% Agree that their work has a small impact (*N*)	6 (11)	12 (18)	2 (1)
Management:			
% Supportive and makes work easier (*N*)	81 (161)	59 (87)	77 (33)
% Not supportive and makes work difficult (*N*)	19 (37)	41 (62)	23 (10)
Facility quality:			
% Unreliable electricity and other services, with supplies you need not always available (*N*)	92 (182)	82 (122)	79 (34)
% Reliable electricity and other services, supplies are always available (*N*)	8 (16)	18 (27)	21 (9)
Training days:			
% 1–5 days (*N*)	46 (92)	26 (39)	21 (9)
% 6–10 days (*N*)	19 (38)	13 (20)	19 (8)
% 11+ days (*N*)	19 (37)	23 (34)	49 (21)
No training (*N*)	16 (31)	38 (56)	11 (5)
Workload:			
% Medium: enough time to complete duties (*N*)	30 (58)	43 (64)	19 (8)
% Heavy: barely enough time to complete duties (*N*)	68 (135)	52 (77)	77 (33)
% Light: more than enough time to complete duties (*N*)	2 (5)	5 (8)	4 (2)
Most motivating factors:^a^			
% Salary (*N*)	12 (11)	6 (4)	0 (0)
% Opportunities to improve health (*N*)	73 (68)	64 (46)	73 (11)
% Management style (*N*)	4 (4)	13 (9)	20 (3)
% Office quality (*N*)	3 (3)	0 (0)	0 (0)
% Training (*N*)	4 (4)	11 (8)	7 (1)
% Workload (*N*)	3 (3)	7 (5)	0 (0)
Most demotivating factors:^b^			
% Salary (*N*)	20 (21)	21 (16)	21 (6)
% Opportunities to improve health (*N*)	8 (8)	9 (7)	11 (3)
% Management style (*N*)	21 (22)	25 (19)	18 (5)
% Office quality (*N*)	11 (11)	5 (4)	7 (2)
% Training (*N*)	10 (10)	16 (12)	11 (3)
% Workload (*N*)	31 (33)	25 (19)	32 (9)
Other:			
% Agree that colleagues often share what they have learnt during training (*N*)	89 (176)	71 (106)	84 (36)
% Agree that work is fairly distributed amongst colleagues (*N*)	83 (165)	78 (116)	84 (36)
% Agree that tasks are often rushed because there is too much work to do (*N*)	78 (155)	68 (102)	74 (32)

a We did not receive a complete response rate for this question. Only answered by 47% (*N* = 93) of HEWs, 48% (*N* = 72) of care providers and 35% (*N* = 15) of non-patient-facing administrative and managerial staff.

b We did not receive a complete response rate for this question. Only answered by 53% (*N* = 105) of HEWs, 52% (*N* = 77) of care providers and 65% (*N* = 28) of non-patient-facing administrative and managerial staff.

### Analysis of preference data—main effects

Table 4 shows MNL results for the main effects models. We found that care providers preferred choosing jobs with a salary 20% above average rather than 20% below average (OR = 1.70, *P*-value = 0.047). Additionally, the odds of choosing a job with average earnings compared with 20% below average influenced the choices of HEWs (OR = 1.43, *P*-value = 0.02) and care providers (OR = 2.00, *P*-value < 0.001). However, choosing a job with a large impact on patient outcomes compared with a marginal impact did not influence the choices of any cadre. A supportive management style was the most preferred job attribute amongst care providers (OR = 3.22, *P*-value < 0.001) and non-patient-facing administrative and managerial staff (OR = 2.96, *P*-value = 0.001) and the second most preferred attribute amongst HEWs (OR = 3.26, *P*-value < 0.001). However, supportive management style was the only attribute that strongly influenced the job choices of non-patient-facing administrative and managerial staff. Good facility quality influenced the job choices of HEWs (OR = 2.42, *P*-value < 0.001) and care providers (OR = 2.69, *P*-value < 0.001). Five days of dedicated training time per year was the most preferred attribute amongst HEWs (OR = 3.45, *P*-value < 0.001) and the third most preferred attribute amongst care providers (OR = 2.67, *P*-value < 0.001). However, 10 days of training per year compared with no training days was associated with disutility amongst care providers (OR = 0.44, *P*-value < 0.001). Similarly, a medium workload over a light workload was associated with disutility amongst HEWs (OR = 0.58, *P*-value < 0.001) and care providers (OR = 0.63, *P*-value = 0.004). However, a job with a heavy workload did not influence the choices of one or more cadre. The odds of choosing their current job over the hypothetical job posts was associated with disutility amongst HEWs (OR = 0.23, *P*-value = 0.005) and care providers (OR = 0.41, *P*-value = 0.030). This indicates that they prefer the hypothetical job posts more to their current post as they were less likely to opt-out.

**Table 4.** MNL main effects results

	HEWs		Care providers		Non-patient-facing staff	
Attributes	OR (CI)	*P*-value	OR (CI)	*P*-value	OR (CI)	*P*-value
Salary:						
20% above average	0.94 (0.54; 1.63)	0.83	**1.70 (1.01; 2.86)**	**0.047**	1.22 (0.55; 2.71)	0.62
Average earnings	**1.43 (1.05; 1.94)**	**0.02**	**2.00 (1.43; 2.79)**	**<0.001**	1.61 (0.87; 2.98)	0.13
20% below average	– (1.00)	–	– (1.00)	–	– (1.00)	–
Impact on patient outcomes:						
Large	1.06 (0.70; 1.61)	0.78	0.99 (0.63; 1.57)	0.973	1.05 (0.49; 2.26)	0.90
Small	– (1.00)	–	– (1.00)	–	– (1.00)	–
Management style:						
Supportive	**3.26 (2.35; 4.52)**	**<0.001**	**3.22 (2.19; 4.73)**	**<0.001**	**2.96 (1.53; 5.73)**	**0.001**
Unsupportive	– (1.00)	–	– (1.00)	–	– (1.00)	–
Facility quality:						
Good	**2.42 (1.81; 3.25)**	**<0.001**	**2.69 (1.92; 3.77)**	**<0.001**	1.09 (0.59; 2.01)	0.79
Unreliable	– (1.00)	–	– (1.00)	–	– (1.00)	–
Training per year:						
5 days	**3.45 (2.16; 5.50)**	**<0.001**	**2.67 (1.46; 4.88)**	**0.001**	1.41 (0.65; 3.09)	0.39
10 days	0.77 (0.44; 1.34)	0.35	**0.44 (0.27; 0.72)**	**0.001**	0.43 (0.16; 1.16)	0.095
No training	– (1.00)	–	– (1.00)	–	– (1.00)	–
Workload:						
Medium	**0.58 (0.44; 0.78)**	**<0.001**	**0.63 (0.45; 0.86)**	**0.004**	0.71 (0.36; 1.40)	0.33
Heavy	0.71 (0.25; 2.03)	0.53	0.74 (0.39; 1.41)	0.363	0.59 (0.15; 2.29)	0.45
Light	– (1.00)	–	– (1.00)	–	– (1.00)	–
Opt-out:						
Yes	**0.23 (0.08; 0.64)**	**0.005**	**0.41 (0.18; 0.92)**	**0.030**	0.47 (0.14; 1.54)	0.21
No	– (1.00)	–	– (1.00)	–	– (1.00)	–
No. of observations	4158		3129		903	
No. of respondents	198		149		43	
Pseudo ${{\bf{\it{R}}}^2}$	0.23		0.20		0.15	

Confidence intervals (CIs) are in parentheses. Standard errors were adjusted for clustering at the facility level, including 80 clusters for care providers, 125 clusters for HEWs and 43 clusters for non-patient-facing administrative and managerial staff. ORs and *P*-value highlighted in bold are statistically significant at either 5% or 1%.

## Discussion

This study examined the job preferences of public sector health workers across Ethiopia. Whilst earning an average salary was found to be important to job choices, non-financial attributes were more important to staff, specifically supportive management, good facility quality and 5 days of training per year. Our findings are consistent with other studies which emphasize the need for a combination of financial and non-financial incentives to increase job satisfaction (Mangham and Hanson, 2008; Huicho *et al.*, 2012; Arora *et al.*, 2020).

One of the key strengths of this study in relation to other studies is that it explores the preferences of a group of health workers of which there has been little or no research (Kolstad, 2011; Jaskiewicz *et al.*, 2012; Mandeville *et al.*, 2014). While understanding the preferences of medical doctors are key to limiting brain drain, HEWs take up around 20% of the recurrent health budget in Ethiopia (Wang *et al.*, 2016), and non-patient-facing administrative and managerial staff are key to implementing QI changes (Tappen *et al.*, 2017). This study finds similar results to other DCE studies in LMICs where it has been found that training, infrastructure and salary are key determinants of job preferences (Hanson and Jack, 2007; Mangham and Hanson, 2008; Kolstad, 2011; Huicho *et al.*, 2012; Abdel-All *et al.*, 2019; Saran *et al.*, 2020). However, many studies have not explored the choice of supportive management, which had the greatest impact across the three cadres.

There are several limitations to this study. First, this study draws out the stated preferences of health workers under hypothetical situations which may not accurately predict their real-life choices, leading to hypothetical bias (Hensher *et al.*, 2005; Quaife *et al.*, 2018). However, Mandeville *et al*. (2016) argue that the revealed preference data are unable to discern the individual effect of each attribute which tends to be affected by multicollinearity. Second, although conducting the same DCE across cadres allows comparison of preferences by cadre, cadre-specific designs may have better reflected the precise factors affecting health workers of different cadres (although we note that no attributes were identified as irrelevant by participants during piloting). Third, because the sample was stratified at cadre level, we were not able to separate out different care provider specialties in this study. Fourth, this study does not include willingness-to-pay estimates, which can provide a useful comparison on how much health workers are willing to be paid for a trade-off between attributes within a job (Mandeville *et al.*, 2014; Lancsar *et al.*, 2017). Fifth, although there were no observed differences between randomly and non-randomly selected woredas, unobserved differences may exist which may negatively affect the generalizability of these results. Finally, the MNL model does not take into account unobserved heterogeneity—the assumption that observations are independent from unobserved effects is arguably restrictive. Mixed logit (MXL) models can ease the IID assumption by allowing for correlation of unobserved effects across individuals. However, there are also some challenges with MXL. For example, MXL models may not produce results that are easy to interpret. The advantage of MNL is its simplicity in estimating models and interpreting results for policymakers, and IIA is considered a reasonable assumption if alternatives are generic (Lancsar *et al.*, 2017). Additionally, MXL models can lead to models being ‘overfitted’, which can reduce its predictive power (de Bekker‐Grob *et al.*, 2012). Furthermore, MXL models require parameter distributional assumptions, and these must come from the analyst and it is difficult to identify a priori which distributions should be assumed for different parameters (de Bekker‐Grob *et al*., 2012; Mandeville *et al.*, 2014). Standard errors were therefore adjusted for clustering at the facility level by allowing for intra-group correlation (Lancsar *et al.*, 2017; StataCorp, 2019).

We recommend that government policymakers consider a combination of financial and non-financial incentives to improve the retention of health workers in Ethiopia. For example, leadership, communication and supportive management training programmes could be implemented to improve the relationship between managers and those managed. In addition, investment in infrastructure to strengthen the quality of hospital facilities may require more macro-level planning between different ministry departments, such as due to increases in revenue, improvements in roads in rural regions to ensure timely supply of drugs, and improvements in supply chain and procurement. Other key policies worth considering are alternative monetary incentives in the event of restricted government budgets, such as tax reduction, transportation allowance or land donation. For example, one study found that these measures improved the retention of health workers in Ethiopia (Dohlman *et al.*, 2019). Finally, improvements in training, mentoring, coaching and other professional development opportunities could deliver positive externalities (Kolstad, 2011), especially as 81% of respondents often shared with colleagues what they had learnt during training.

Our a priori hypothesis that ‘opportunities to improve patient outcomes’ would be important to choices was not shown to be correct. However, almost 70% of DCE respondents chose this attribute as the factor that most motivates them. This may be due to social desirability or response bias in direct questioning, which participants feel can be hidden from the interview in DCE choices which are based on trading-off multiple attributes. It is worth exploring this further in future studies. Furthermore, a study, conducted in the early years of HEP, reported regional variations in many working conditions of HEWs and noted that there were challenges in harmonizing aspects such as the staffing pattern, HEW work schedules and relationship with the community, between regions. Other aspects such as the stock of medicines available at health centres was also found to be different, mostly favouring richer regions with better health infrastructure like Tigray over health posts in Oromia (Teklehaimanot *et al.*, 2007). Due to the small sample size, it was not possible to explore heterogeneity by region—however, other studies have also shown that Tigray has traditionally performed better on health indicators compared with SNNPR and so we expect variation in health worker job preferences between regions (Arora *et al.*, 2020).

## Conclusions

This study used a DCE to estimate which aspects of a job are most influential to health worker choices in Ethiopia, including HEWs, care providers such as nurses and midwives, and non-patient-facing administrative and managerial staff. A multinomial logistic regression model estimated the importance of six attributes to respondents: salary, training, workload, facility quality, management and opportunities to improve patient outcomes. We found that non-financial factors were important to respondents from all three cadres: e.g., a supportive management style was found to be one of the most important attributes across all cadres in Ethiopia including HEWs, care providers and non-patient-facing administrative and managerial staff. Whilst earning an average salary also influenced the job choices of HEWs and care providers, other attributes were more important including good facility quality and 5 days of training per year. This shows that a combination of financial and non-financial incentives should be considered to motivate health workers in Ethiopia.

## Data Availability

The data sets generated and analysed in the study are available on reasonable request made to the corresponding author.

## Abbreviations

CI - Confidence intervals.

DCEs - Discrete Choice Experiments.

ETB - Ethiopian Birr.

HEP - Health Extension Programme.

HEWs - Health Extension Workers.

IID - Independently and Identically Distributed.

LMICs - Low- and Middle-Income Countries.

MNL - Multinomial Logit.

OR - Odds Ratios.

QI - Quality Improvement.

SNNPR - Southern Nations, Nationalities, and Peoples’ Region.

USD - US Dollar.
